# Sucupira as a Potential Plant for Arthritis Treatment and Other Diseases

**DOI:** 10.1155/2015/379459

**Published:** 2015-11-03

**Authors:** Jaqueline Hoscheid, Mara Lane Carvalho Cardoso

**Affiliations:** Postgraduate Program in Pharmaceutical Sciences, State University of Maringá, Avenida Colombo 5790, 87020-900 Maringá, PR, Brazil

## Abstract

Trees of the genus *Pterodon*, commonly known as “sucupira-branca” or “faveira,” are native to central Brazil. The *Pterodon* fruits are traditionally used in ethnomedicine as an infusion, in small doses, and at regular time intervals as an antirheumatic, anti-inflammatory, tonic, and depurative agent. The various compounds present in the *Pterodon* class are, generally, water-insoluble and derived from the fusion of high-molecular weight pentacarbonate units. Scientific research has shown that the major compounds isolated from *Pterodon* species are linear and/or tetracyclic diterpenes with vouacapane skeletons that partly underlie the pharmacological activities of the fruit-derived oil. Material from *Pterodon* species has several biological properties, such as analgesic, anti-inflammatory, and anticancer effects. Therefore, recent studies have sought to microencapsulate these extracts to protect them from potential chemical degradation and improve their water solubility, ensuring greater stability and quality of the end products. This review presents a succinct overview of the available scientific evidence of the biological activity and toxicity of *Pterodon* species in addition to other important aspects, including phytochemical and technological features.

## 1. Introduction

The fruits of the genus* Pterodon*, known as “sucupira,” are sold in popular markets and are used in traditional medicine where they are ingested in small amounts and at regular time intervals for the treatment of rheumatism, sore throat, and respiratory disorders (bronchitis and tonsillitis), in addition to their anti-inflammatory, analgesic, purifying, tonic, and hypoglycemic activities [1–4].

The medicinal properties of the plants from the genus* Pterodon* were investigated at the same time when the first active metabolites were isolated, based on folk medicine where wine, cachaça, or boiling water (tea) infusions of the plants are used [5, 6]. Studies have shown that linear terpenes, geranylgeraniol derivatives, and tetracyclic diterpenes, which have a vouacapane or vinhaticane skeleton, partly underlie the pharmacological activities of the extracted* Pterodon* fruit oil [6–12]. To assess the toxicity, the biochemical, hematological, and clinical parameters were determined in previous studies [11, 13]. The increasing use of the herbal* Pterodon* drugs aroused concern about their effectiveness. Therefore, several researchers have sought to preserve the characteristics of the plant extracts, since improper processing of the plants may result in degradation of the active compounds and, therefore, the loss of pharmacological activity [14–16]. This review presents a scientific overview of “sucupira” trees with reference to their ethnobotanical uses, phytochemistry, biological activity, and toxicity as well as the technological aspects of development phytotherapeutics.

## 2. The Genus* Pterodon*

According to the list of Brazilian flora species, the genus* Pterodon* (Fabaceae/Leguminosae) comprises four species native to Brazil:* P*.* abruptus* Benth.,* P*.* appariciori* Pedersoli,* P*.* pubescens* Benth., and* P*.* emarginatus* Vog. (synonymous with* P*.* polygalaeflorus* Benth.) [17] which are commonly known as “faveira,” “sucupira,” “sucupira-branca,” “fava-de-sucupira,” or “sucupira-lisa” [18]. The plants are distributed disjunctively and mixed populations have not been observed, which may indicate that certain soil characteristics are a limiting factor in the plants' development [19]. In general, these species are aromatic, native trees growing 6–18 m tall, and are distributed throughout the central region of Brazil, especially in the Cerrado (Goiás, Minas Gerais, São Paulo, Tocantins, Mato Grosso, and Mato Grosso do Sul) and the semideciduous forest of the Paraná Basin [20, 21]. Despite their slow growth,* Pterodon* species are tolerant to direct sunlight and low soil fertility and are therefore important in mixed reforestation for the recovery of degraded areas [22].

**Figure 1:**
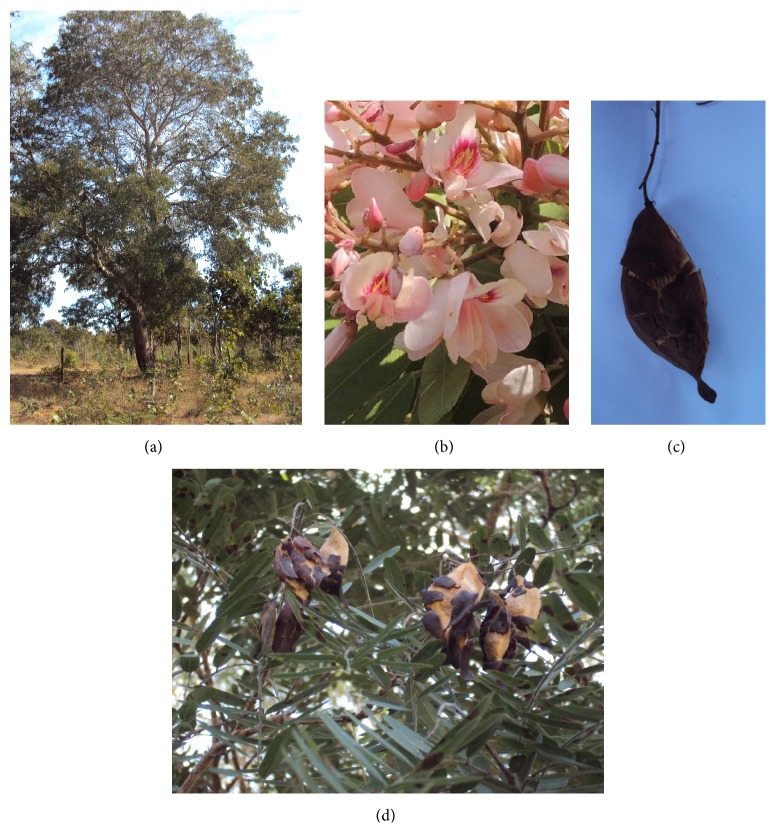
Photographs of the* Pterodon pubescens* tree in habitat (a), flowers (b), fruit (c), and a fruiting branch (d). Photographs courtesy of Dr. Mara Lane Carvalho Cardoso.

*P. pubescens* has pinnate leaves containing 20–36 leaflets. Its wood is often used in naval and civil constructions because of its heavy, compact tissue and long durability even when in contact with soil and moisture (Figure 1(a)) [23]. The flowers have a whitish or rosy coloring and are arranged in axillary and apical panicles (Figure 1(b)) [18]. The fruit matures from June to July when the plant is almost completely stripped of its foliage [20]. The fruit pods are of the rounded samara type, indehiscent and winged, containing only a heavily protected seed within a fibrous, wooden capsule that is wrapped externally by a bitter, oily substance in a sponge (Figures 1(c) and 1(d)) [18, 20]. The fruits contain a core endosperm enveloped in peripheral cells, with rich and dense lipid substance content [22]. The fruits can either be collected directly from the trees in the season when the fruits start falling or after they have fallen from the trees. They may be used directly for sowing purposes or opened for removal of the seed before planting. One kilogram of fruit material contains approximately 1200 fruits [20].

*P*.* pubescens* shares several similarities with* P*.* emarginatus* and their shared common name is often the cause for confusion. However,* P*.* emarginatus* grows in regions north of those of* P*.* pubescens* in Brazil and the main differences between the two subtypes are the blue-violet flowers and the length of* P*.* emarginatus* trees, which can be up to 15 m, while* P*.* pubescens* grows to a height of 5–10 m [20]. The appropriate way to differentiate between the* Pterodon* species is by evaluating the anatomical structure of the extrafloral nectaries, which exist in the rachis and are located under the insertion of each petiole. The basic difference between these two subspecies is the density of the pubescence, which is always greater in* P*.* pubescens* [19].* P*.* pubescens* fruits are also often confused with those of* Bowdichia virgilioides* Kunth., a medium-sized tree found in the tropical rainforests of South America and known in folk medicine as “sucupira-preta” [24, 25].

## 3. Phytochemistry

The improvement of the purification and isolation methods using mass and ultraviolet spectrometry and infrared and nuclear magnetic resonance techniques has allowed the elucidation of complex structures in plant materials [26, 27]. Phytochemical studies on the genus* Pterodon* have demonstrated the presence of isoflavones, sesquiterpenes, and diterpenes in its fruit oil [1, 28–30]; isoflavones and triterpenes in the wood [31, 32]; alkaloids, saponins, glycosides, and steroids in the bark of the trees [24, 33]; and steroids, sesquiterpenes, isoflavones, and saponins in the leaves [34, 35].

**Figure 2:**
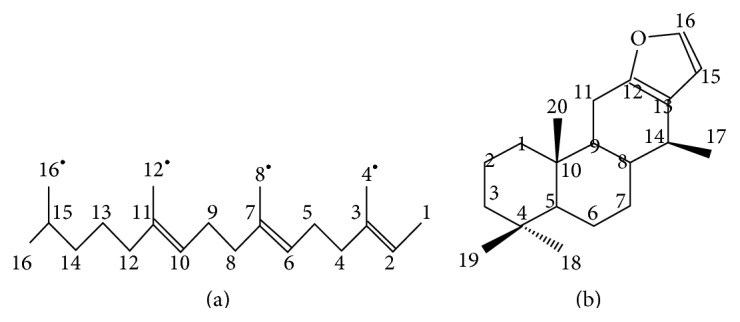
Basic structure of diterpenes: (a) linear diterpene and (b) vouacapane skeleton.

The main isolated compounds of these species are diterpenes, which belong to the group of terpenes or terpenoids and constitute the largest class of secondary metabolites. The various substances of this class are, generally, water-insoluble and are derived from the fusion of pentacarbon units, which have a branched isopentane backbone. These substances are classified according to the number of units they contain, which may have extensive metabolic changes. The 10-, 15-, and 20-carbon terpenes are called monoterpenes, sesquiterpenes, and diterpenes, respectively. The largest terpenes include the triterpenes (30-carbon), tetraterpenes (40-carbon), and polyterpenoids [36]. The essential constituents of the* Pterodon* species are linear and/or tetracyclic diterpenes with a vinhaticane or vouacapane skeleton (Figures 2(a) and 2(b)) [37].

Early identification studies of the compounds present in the genus* Pterodon* were performed in the 1960s. Mors et al. [38] identified the diterpenes 14,15-epoxygeranylgeraniol and geranylgeraniol in the oil of* P. pubescens* fruits, which proved to be efficient as chemoprophylactic agents in schistosomiasis. Since then, a wide variety of compounds was isolated.  Table 1 summarizes the main molecules published in the literature to date.

Since the discovery of furan diterpenes, especially 6*α*,7*β*-dihydroxyvouacapan-17*β*-oic acid, they have been used as a prototype for the synthesis of novel molecules with different biological activities in several studies [39–41]. It is believed that the biological activities of the oil from the seeds of the species of the genus* Pterodon* are directly related to the furan diterpenes content [9].

Based on these findings, it becomes clear that the compounds present in the fruits of the species of the genus* Pterodon* are highly similar, which suggests that these compounds may have similar biological and pharmacological activities. However, botanical and chemical similarities often hinder interspecies differentiation.

Environmental and genetic factors may affect the development of several plant species, in particular that of aromatic species. For example, the qualitative and quantitative variations of secondary metabolites [42] that directly influence the quality of the medicinal plant are related to domestication of the species, as was observed by Alves et al. [43] when evaluating the essential oil of* P*.* emarginatus* fruits collected from five different locations of the Cerrado.

## 4. Physicochemical Properties

A few studies have characterized the physicochemical properties of the oil extracted from species of the genus* Pterodon*. Hoscheid et al. [14], using gas chromatography-mass spectrometry (GC-MS), identified the predominant diterpene observed in the chromatographic profile of the hexane fraction of the ethanol extract of* P. pubescens* as 14,15-epoxygeranylgeraniol (retention time: 34.41 min.). According to the authors, compounds with a retention time between 42.21 and 48.02 min correspond to vouacapane derivatives present in the analyzed fraction.

Reinas et al. [16] have used thermal and infrared analysis methods to analyze the oilseed fraction of* P*.* pubescens* fruits and found a 0.11% residual mass at 500°C by differential scanning calorimetry (DSC) and thermogravimetric analysis (TGA). The oil absorption spectra in the infrared region (FT-IR) showed bands at 1745 cm^−1^ (assigned to the C=O stretching of acetates), at 1244 cm^−1^ (indicating the asymmetric C-O stretching of acetates), at 1017 cm^−1^ (denoting asymmetric stretching of C-O-C esters), and at 732 cm^−1^ (reflecting a furan ring). Furthermore, Fourier transform Raman (FT-Raman) spectroscopy analysis showed a peak at 1673 cm^−1^, reflective of the ester group of vouacapanes [16]. These data are useful for future inter- or intraspecies comparison of the physicochemical characteristics of the fruits' oilseed fraction.

## 5. Traditional Use and Biological Activities

### 5.1. The Use in Traditional Medicine

Fruits from the species of the genus* Pterodon* are sold on popular markets and are used in traditional medicine in small doses and at regular time intervals for the treatment of rheumatism, sore throats, and respiratory disorders (bronchitis and tonsillitis). These therapeutic effects are owing to the fruits' anti-inflammatory, analgesic, purifying, tonic [1, 3, 55], and hypoglycemic activities [2]. Fruit infusions can also be applied externally against acne and skin patches [3]. The active metabolites of* Pterodon* species were isolated and their medicinal properties were investigated based on folk medicine where wine, cachaça, or boiling water (tea) infusions of the plants are used [5].

### 5.2. Antinociceptive Activity

The antinociceptive potential of the fruits of* Pterodon* species has been demonstrated for both the oil extract and its fractions as well as for the isolated diterpenes. The ethanol extract of* P*.* polygalaeflorus* demonstrated a significant analgesic effect at a dose of 200 mg/kg body weight (b.w.), as assessed by the inhibition of contractions induced by acetic acid, dextran, and formalin* in vivo* animal models, explaining its traditional use in the treatment of respiratory diseases in the northeastern regions of Brazil [56].

The antinociceptive properties of the ethanol extract and those of the dichloromethane and hexane fractions (130 mg/kg b.w., p.o.) from* P*.* pubescens* seeds were also shown to be promising in the treatment of pain disorders, with the effect attributable to the vouacapane derivatives as identified in the same study [45]. These findings were supported by the effect of the intragastric infusion of the oily extract of the same species (10–100 mg/kg b.w.) in models of acute and chronic pain, in which the analgesic potential was significant in both models, inhibiting the mechanical and thermal hyperalgesia in postoperative and complex regional pain syndrome [48].

A comparative study on the ethanolic, hexane, and dichloromethane extracts of* P*.* polygalaeflorus* fruits demonstrated an increased antinociceptive effect of the hexane extract (100% inhibition) achieved at lower doses (0.5 mg/kg b.w., p.o.) compared to that observed for isolated vouacapane derivatives in other studies. This indicates the existence of a synergistic effect of the compounds present in the extract. Phytochemical analysis identified that 90% of the material was composed of sesquiterpene hydrocarbons, oxygenated sesquiterpenes, and furan diterpenes [30].

The central and peripheral analgesic effect of the essential oil and the hexane, methanol, and butanol fractions of* P*.* emarginatus* seeds (100, 300, and 500 mg/kg b.w.) were first demonstrated by Dutra et al. [57] by the significant reduction of abdominal writhing and the number of paw licking events, and the increase in the latency in the hot plate test. The potential involvement of opioid peptides in the mechanism of action underlying 6*α*-7*β*-dihydroxyvouacapan-17*β*-oate extracted from* P*.* polygalaeflorus* was investigated in the acetic acid-induced writhing response mouse model and in the carrageenan-induced inflammatory hyperalgesia rat model. Vouacapane caused dose-dependent analgesia in both models when administered orally, subcutaneously, and intraperitoneally, showing that the release of endorphins may be involved in the mechanism underlying diterpene's analgesic effect [58]. A synergistic analgesic effect was observed when clonidine or dopamine was associated with vouacapane, indicating, at least partly, the activation of the catecholaminergic system [59].

Other compounds, such as geranylgeraniol and 6*α*,7*β*-dihydroxyvouacapan-17*β*-oate methyl ester from* P*.* pubescens* and 6*α*,7*β*-dihydroxyvouacapan-17*β*-oic acid from* P*.* emarginatus*, were evaluated separately to verify their contributions to the antinociceptive activity of the crude extracts [10, 51, 60]. Geranylgeraniol and 6*α*,7*β*-dihydroxyvouacapan-17*β*-oate methyl ester demonstrated activities related to the inhibition of vanilloid receptors (VR1), glutamate peripheral receptors [10], and the synthesis and release of serotonin [60]. The compound 6*α*,7*β*-dihydroxyvouacapan-17*β*-oic acid showed a predominant peripheral analgesic effect, even when used alone [51]. In addition, a synergistic effect was observed among the isolates and other metabolites present in the extracts, based on the fact that the crude extract (300 mg/kg b.w., p.o.), which contained small amounts of all compounds studied, had statistically identical effects when administered at the same dose as that of the pure compounds tested separately [10].

A more recent study indicated that the antinociceptive activity of the hydroethanolic extract from* P*.* emarginatus* leaves (500 and 1000 mg/kg b.w.) could have been related to the presence of flavones and hederagenin derivatives, which were identified as the major constituents of the extract [35].

### 5.3. Anti-Inflammatory Activity

The first studies to elucidate the anti-inflammatory activity of the “sucupira” fruit monitored the performance of mice with type II collagen-induced arthritis [61, 62]. A significant reduction in the arthritis index scores was achieved by preventive treatment, starting 20 days before the arthritis induction with daily administration (5 mg/kg b.w., p.o.) of the hydroalcoholic extract from* P*.* pubescens* seeds. These results provide a scientific basis for the popular use of the seed infusions to treat arthritis. Carvalho et al. [7] demonstrated that the hexane extract (500 mg/kg b.w., p.o.) from* P*.* emarginatus* fruits had anti-inflammatory activity and could inhibit neutrophil migration to the peritoneal cavity and granulomatous tissue formation* in vivo*. The authors suggested that the anti-inflammatory activity of the extract could be related to the presence of terpene compounds. Others demonstrated the antiedematogenic activity of the ethanol extract and of the fractions of* P*.* pubescens* [8], as well as the reduction (approximately 68%) in carrageenan-induced edema by the hydroethanol extract from* P*.* polygalaeflorus* fruits (200 mg/kg, i.p.) [56].

Eccentric muscle contractions, arising from acute exercise induced by functional electrical stimulation, may result in muscle trauma and, consequently, initiate an inflammatory process. A protective effect of the hexane extract from* P*.* emarginatus* fruits was observed when administered before and after acute exercise (498 mg/kg b.w., p.o.), confirming the high activity of this oil infusion and, hence, explaining its use against inflammatory processes in folk medicine [63].

Cardoso et al. [5] evaluated the effect of the ethanol extract from* P*.* pubescens* fruits on the suppression of T and B lymphocytes and on nitric oxide production by macrophages and provided evidence that the extract could suppress the cellular and humoral immune responses in healthy rats at lower concentrations (10^−2^ mg/kg b.w.) than dexamethasone. The demonstrated immunomodulatory activity is a desired effect because of the fruits' potential application in the treatment of patients with immunoinflammatory or chronic autoimmune diseases. Research conducted with the diterpene 6*α*,7*β*-dihydroxyvouacapan-17*β*-oic acid (50 mg/kg b.w., p.o.), isolated from* P*.* emarginatus*, showed a significant inhibition of edema in carrageenan- and prostaglandin E2-induced edema models, indicating that this vouacapane derivative is one of the agents responsible for the anti-inflammatory action of the extract from* P*.* emarginatus* species [51].

Recently, it was demonstrated that the hexane fraction of the ethanol extract from* P*.* pubescens* fruits (250 mg/kg/day b.w., p.o.) had an inhibitory effect on acute inflammation in the carrageenan-induced rat model of pleurisy and on chronic inflammation in the complete Freund's adjuvant-induced arthritis rat model [11]. In the same year, the ethanolic, hexane, and dichloromethane extracts from* P*.* polygalaeflorus* fruits were assessed for their anti-inflammatory capacity. The hexane extract showed a greater potential for reducing signs of inflammation (redness and vasodilation), accompanied by a reduction in the total number of leukocytes in the exudate (0.01–1 mg/kg b.w.) and reduced tissue thickening (1 mg/kg b.w.), in the* in vivo* air pouch model. Furthermore, it inhibited nitrite production in RAW 264.7 cells (0.1–80 *μ*g/mL), demonstrating high anti-inflammatory activity [30]. Finally, trials using* P. emarginatus* essential oil (100 mg/kg b.w., p.o.), which is rich in aromatic terpenes and phenylpropanoids, showed significant attenuation of neurological signs, with reduced and limited development of autoimmune encephalomyelitis by the modulation of the Th1/Treg cell balance [12].

Overall, these results encourage the development of novel research on* Pterodon* species for the development of standardized extracts and formulations with anti-inflammatory potential.

### 5.4. Antioxidant Activity

Paula et al. [63], in an* in vivo* study using the hexane extract of* P*.* emarginatus* fruits, showed that administration of the extract (498 mg/kg b.w., p.o.) before and after functional electrical stimulation abolished most of the oxidative and nitrosative processes in organs after acute exercise, suggesting a high antioxidant activity of the hexane extract. The phenolic and flavonoid content and the antioxidant activity of essential oil and of butanolic, methanolic, hexane, and ethyl acetate extracts from* P. emarginatus* fruits have also been investigated. The use of methanol and butanol as extractor agents increased the levels of the phenolic constituents and, consequently, demonstrated the highest 2,2-diphenyl-1-picrylhydrazy (DPPH) radical scavenging activity (IC_50_: 10.15 and 18.89 *μ*g/mL for the methanol and butanol extract, resp.) [64].

### 5.5. Antimicrobial Activity

The chemoprophylactic capacity of the isolated compound 14,15-epoxygeranylgeraniol [38], of oleaginous extracts [38, 65, 66], and of the fractions composed of diterpene derivatives [28] from* P*.* pubescens* fruits in schistosomiasis has been studied for several years and by several researchers. Research evaluating the larvicidal activity against* Aedes aegypti* of the hexane extract (IC_50_: 23.99 *μ*g/mL) of* P*.* polygalaeflorus* fruits and furan diterpenes (IC_50_: 14.69–50.08 *μ*g/mL) isolated from these fruits were shown to be promising agents for future repellent formulations [53, 67]. Furthermore, the trypanocidal potential of the geranylgeraniol, hexane, dichloromethane, and ethyl acetate fractions and of the ethanol extract of* P*.* pubescens* was investigated [68]. Both the hexane fraction, which was composed of geranylgeraniol, and isolated geranylgeraniol inhibited the proliferation of intracellular amastigotes at concentrations that did not affect mammalian host cells (22 and 15 *μ*g/mL, resp.). Moreover, they were three times more active than the other fractions, mainly acting on the mitochondria of the epimastigotes and trypomastigotes of* Trypanosoma cruzi*.

Dutra et al. [69] showed that the essential oil from* P*.* emarginatus* seeds inhibited the growth of* S*.* aureus* (minimum inhibitory concentration (MIC): 2.5 mg/mL). The hexane and butanol fractions from* P*.* emarginatus* seeds had significant* in vitro* activity against* Leishmania amazonensis* (IC_50_: 50.06 and 46.65 *μ*g/mL, resp.). According to the authors, these results indicate that bioactive molecules found in* P*.* emarginatus* seeds can be used as prototypes for the development of new drugs and/or as sources of raw pharmaceutical materials with antimicrobial and leishmanicidal activities.

The activity of the ethanol extract from* P*.* polygalaeflorus* bark against* Staphylococcus aureus*,* Micrococcus flavus*,* Bacillus cereus*,* Bacillus subtilis*,* Salmonella enteritidis*,* Escherichia coli*,* Pseudomonas aeruginosa*,* Proteus mirabilis*,* Serratia marcescens*,* Mycobacterium phlei*,* Mycobacterium smegmatis*, and* Mycobacterium fortuitum* and against the yeasts* Candida albicans* and* Candida krusei* was tested and was significant against* Mycobacterium phlei* only [70]. The evaluation of the antimicrobial activity of* P*.* emarginatus* bark showed a different outcome since the use of its ethanol extract against Gram-positive and Gram-negative bacteria and against the fungus* Candida albicans* was effective against* Rhodococcus equi*,* Micrococcus luteus*,* Micrococcus roseus* (MIC: 0.18 mg/mL),* Serratia marcescens*,* Pseudomonas aeruginosa* (MIC: 0.37 mg/mL),* Enterobacter cloacae*, and* C*.* albicans* (MIC: 0.74 mg/mL) [33].

However, when the extract was prepared using Brazilian cachaça as extractor liquid and was tested against* Bacillus subtilis*,* Staphylococcus aureus*,* Escherichia coli*,* Pseudomonas aeruginosa*,* Candida albicans*,* Candida parapsilosis*, the promastigote form of* Leishmania amazonensis*, and poliovirus, it did not show activity at concentrations lower than 1000 *μ*g/mL. Several plant species in the Brazilian Cerrado are widely used in ethnomedicine, though the safety and efficacy of medicinal plants used in communities are not always known. The risk of toxicity due to the inappropriate use of herbal drugs should be conveyed to the population [71]. Nevertheless, taking into account that the alcohol content does not exceed 40% in Brazilian cachaça and that sesquiterpenes and diterpenes are insoluble in water, the choice of liquid extractor could directly affect the extraction of active compounds and, consequently, limit both the antimicrobial activity and the pharmacological toxicity profile of the extracts.

### 5.6. Anticancer Activity

The inhibition of hepatic preneoplastic lesions and cell proliferation by purified geranylgeraniol renders this compound a promising chemopreventive agent against hepatocarcinogenesis [72]. Several studies using* P*.* pubescens* fruits have investigated their antiproliferative activity against various cancer cell lines. Studies using isolated furan diterpenes, including 6*α*-acetoxy-7*β*-hydroxyvouacapan, 6*α*,7*β*-dihydroxyvouacapan-17*β*-oate methyl ester, and 6*α*,7*β*-dihydroxyvouacapan-17*β*-methylene-ol, against prostatic cell lines [9] and hexane extract subfractions containing vouacapan-6*α*,7*β*,14*β*,19-tetraol against human melanoma cell lines [46] showed improved antiproliferative effects and a lower toxicity than that found for doxorubicin.

The furan diterpene 6*α*,7*β*-dihydroxyvouacapan-17*β*-oic acid isolated from* P*.* polygalaeflorus* showed promising results as an antiproliferative agent against human cancer cell lines. Likewise, the semisynthetic derivatives 6*α*-hydroxyvouacapan-7*β*,17*β*-lactone and 6-oxovouacapan-7*β*,17*β*-lactone had effective and selective cytotoxicity against the ovarian cancer cell lines NCI-ADR/RES and OVCAR-03 and erythromyeloblastoid leukemia cells (K562), respectively [41].

The effect of a terpenoid-rich subfraction of purified* P*.* pubescens* was assessed in the K562 (human myeloid leukemia) and Jurkat (human T-cell acute leukemia) cell lines and was found to be significantly cytotoxic by arresting cell cycle progression and inducing apoptosis [73]. Similarly, Pereira et al. [47] found that subfractions of the hexane extract of* P*.* pubescens* containing epoxyfarnesol, geranylgeraniol, and vouacapane derivatives (methyl 6*α*-acetoxy-7*β*-hydroxyvouacapan-17*β*-oate and 6*α*,7*β*-dihydroxyvouacapan) were able to inhibit the activation of signaling pathways involved in chronic myeloid leukemia cell proliferation and to stimulate cell cycle arrest and apoptosis.

Essential oil of* P*.* emarginatus* was active against rat glioma (C_6_), human melanoma (MeWo), mouse colon carcinoma (CT_26_.WT), human breast cancer (MDA), human lung carcinoma (A_549_), mouse melanoma (B_16_-F_1_), hamster ovary cell (CHO-K1), and hamster kidney fibroblast cell lines (BHK-21) with IC_50_ of 24.9–47 *μ*g/mL. In an* in vitro* cell viability assay, treatment with the essential oil showed higher viability of peripheral blood mononuclear cells in comparison with doxorubicin treatment. In summary, these data show that essential oil is less cytotoxic against normal cells but has increased cytotoxicity against cancer cells. Oil analysis revealed the presence of *β*-elemene,* trans*-caryophyllene, *α*-humulene, germacrene D, bicyclogermacrene, and spathulenol in concentrations higher than 5% [29].

The ethanol, hexane, and dichloromethane extracts and 14,15-epoxygeranylgeraniol isolated from* P*.* emarginatus* demonstrated inhibition of cell proliferation and viability of the human glioblastoma cell line U87MG [6]. These results support the interest in developing new anticancer therapeutics in the near future.

### 5.7. Muscle Relaxant Activity

The ethanol extract from* P*.* polygalaeflorus* fruits showed bronchodilator activity in isolated guinea-pig trachea (EC_50_: 1.7 mg/mL), supporting its use in respiratory diseases [56]. Similar results were observed for the essential oil of these fruits (1300 *μ*g/mL), which inhibited contractions triggered by electromechanical coupling [42]. In addition, the essential oil of* P*.* polygalaeflorus* and its main constituent *β*-caryophyllene were shown to have antispasmodic activity and to cause muscle tone relaxation in the isolated rat ileum (IC_50_: 394.35 and 68.65 *μ*g/mL, resp.); their inhibitory effect on intestinal contractility was primarily mediated through an intracellular mechanism [54].

### 5.8. Hypoglycemic Activity

There are few reports on the hypoglycemic effect of extracts from* Pterodon* species. The hypoglycemic activity of tea brewed from the leaves and fruits of* P*.* emarginatus* was described in a study on the traditional use of plants in communities in the Alto Paraguay Bay and Guapore Valley in Mato Grosso in Brazil [2, 74]. Marked changes in blood glucose levels (18.36% decrease) were observed* in vivo* after daily administration of the hexane fraction of* P*.* pubescens* fruits (250 mg/kg b.w., p.o.) for 21 days [11].

### 5.9. Lipolytic Activity

Oral administration of the hexane fraction of* P*.* pubescens* fruits in rats (250 mg/kg/b.w./day, p.o.) for 21 days greatly reduced total cholesterol (34.18%) and triglyceride levels (41.63%) compared with those of the control group, without causing changes in water and food consumption. To the best of our knowledge, cholesterol and triglyceride level-lowering effects of extracts from* Pterodon* species in animals have not been shown in studies previous to the one conducted by Hoscheid et al. [11].

## 6. Toxicity

As important as the activity is the proof of the absence of toxicity. Sabino et al. [75] showed that the extracted oil fraction from* P*.* pubescens* seeds did not induce acute toxic effects, mutagenicity, and/or cytotoxicity in healthy animals after oral administration of doses significantly higher (8 g/kg b.w.) than those ingested by humans. The absence of acute toxicity was also observed for the hexane fraction from* P*.* pubescens* fruits at a dose of 1 g/kg b.w. [11].

Nevertheless, in mice with type II collagen-induced arthritis, the hydroethanolic extract of* P*.* pubescens* is therapeutically effective only after several days of treatment, which could result in subacute toxicity in the animals. To verify this, Pinto Coelho et al. [13] determined the biochemical, hematological, and clinical parameters and performed anatomical and histopathological analysis of the organs of animals treated with the hydroethanol extract of* P*.* pubescens* for 28 days (5 mg/kg b.w./day). The authors noted that in addition to reducing the severity of the disease the treatment did not induce any detectable toxic subacute side effects. Similarly, after daily treatment with the hexane fraction of* P*.* pubescens* (250 mg/kg b.w., p.o.) for 21 days, no anatomical, histological, biochemical, and hematological changes were found in the complete Freund's adjuvant-induced arthritis rat model [11].

In contrast, as described in a case report [76], an outbreak of bovine poisoning in Goiás in Brazil causing illness in 84 animals and the death of seven of them was due to the consumption of the leaves and fruits of* P*.* emarginatus*. A marked elevation of serum bilirubin levels and the activity of serum alanine and aspartate aminotransferase (ALT and AST, resp.) and gamma glutamyltransferase (GGT) was found. Macroscopic changes, such as hepatomegaly and necrosis of multifocal areas in the liver, heart, spleen, lungs, skeletal intercostal muscles, and chest, as well as bleeding were also observed. At the microscopic level, degeneration of the liver and kidneys, hepatocellular necrosis, and biliary hyperplasia were observed.

## 7. Development of Phytotherapeutics Containing Oil from* Pterodon* Species

Biodiversity, together with the traditional knowledge of communities of popular medicine and the science underlying the biological sciences, has driven the technological development of plant products. Early studies seeking to develop a herbal medicine using extracts from* P*.* pubescens* were established in the 1980s and 1990s, which followed the earlier finding of a chemoprophylactic effect on schistosomiasis [38]. Different soap formulations were developed and incorporated with the essential oil obtained by the hexane percolation of fruit. The protective efficacy rates ranged from 0% to 100% depending on the formulation. In addition, preliminary studies on irritation and toxicity were promising, showing that these formulations had potential in the prevention of schistosomiasis [65, 66].

One of the methods of using* P*.* emarginatus* to treat rheumatic pains and burns in folk medicine is the use of poultices prepared by steeping the fruits [77]. To prove the activity of this method, the essential oil and the hexane fraction obtained from* P*.* emarginatus* fruits were incorporated in a cream, and the cream's effect on the healing of skin wounds was tested in rabbits. Both creams had significant anti-inflammatory activity, showing a reduction in the number of inflammatory cells and an increase in the number of fibroblasts and blood vessels, explaining its effect and use in traditional medicine [50].

One of the current trends in the manufacture of herbal medicines to ensure the attainment of the desired therapeutic effect is based on the use of standardized dried extracts [78]. Spray-drying the ethanolic extract from* P*.* emarginatus* using colloidal silicon dioxide prevented thermal degradation and approximately doubled the terpene content, in line with the increase in the analgesic potential of the dry extract [52]. These results might suggest that the technological process used to transform the oil from* P*.* emarginatus* fruits in the dry extract was appropriate since it maintained the quality and therapeutic activity at the level described for plant material.

During the transformation of herbal plant material, the chemical and pharmacological integrity needs to be preserved to ensure a constant composition and therapeutic reproducibility [79]. Therefore, Hoscheid et al. [14] isolated the isomers methyl 6*α*-hydroxy-7*β*-acetoxyvouacapan-17*β*-oate and methyl 6*α*-acetoxy-7*β*-hydroxyvouacapan-17*β*-oate from* P*.* pubescens* fruits and validated a methodology to quantify the compounds by GC-MS using selected ion monitoring. The analyses were carried out using a system equipped with a HP-5 capillary column and temperature program at 100–270°C. The method proved to be precise, reproducible, accurate, and robust. Furthermore, vouacapanes were found to be stable in solution for more than 12 days.

Among the alternatives to preserve the plant material characteristics, microencapsulation is superior [79]. Servat et al. [15] microencapsulated* P*.* pubescens* extract and the isomer mixture of methyl 6*α*-hydroxy-7*β*-acetoxyvouacapan-17*β*-oate and 6*α*-acetoxy-7*β*-hydroxyvouacapan-17*β*-oate in maltodextrin and gum arabic using spray-drying. The results of the accelerated stability study and monitoring of the antinociceptive assays demonstrate that microencapsulation is a useful alternative to increase the shelf life, preserve the contents and to solve shortcomings such as low water solubility.

Another microencapsulation method, complex coacervation, was used for the oilseed fraction of the ethanol extract from* P*.* pubescens* fruits in which the use of a polymeric system consisting of alginate/chitosan with medium- or low-molecular-weight chitosan and a deacetylation degree greater than 75% was evaluated. The use of the alginate/low-molecular-weight chitosan polymer system with a mean polymer diameter of 0.5885 *μ*m and the highest encapsulation efficiency proved to be most promising. According to the authors, this system has the potential to mask the flavor of the extract and to protect it from possible chemical degradation [16].

## 8. Conclusion

The common constituents of the fruits of the species of the genus* Pterodon* are linear diterpenes, geranylgeraniol derivatives, and/or tetracyclic diterpenes with vouacapane skeletons. Several other compounds have been isolated from different plant parts, such as flavonoids, saponins, and sesquiterpenes from the leaves. However, the fruit is the main plant material used. Extracts from* Pterodon* species have several biological properties, such as analgesic, anti-inflammatory, and anticarcinogenic effects. Current* in vitro* and* in vivo* studies confirm these activities and seek to develop phytotherapeutics using methods that preserve the contents of the plant material and resolve solubility issues. In addition, toxicity assessments and observations that were conducted during biological test assays indicate that therapeutics made from* Pterodon* species are safe when taken at the recommended doses.

## Figures and Tables

**Table 1:** Phytochemical investigations of *Pterodon* sp.

Part used	Compound	Reference
*Pterodon pubescens*		

Fruits	14,15-Epoxygeranylgeraniol	[38]
	Geranylgeraniol	[38]
	16-Epoxygeranylgeraniol	[44]
	16,17-Epoxygeranylgeraniol	[44]
	6*α*-Hydroxy-7*β*-acetoxyvouacapan-14(17)-ene	[45]
	7*β*-Acetoxyvouacapane	[45]
	Methyl 6*α*,7*β*-dihydroxyvouacapan-17*β*-oate	[45]
	Methyl 6*α*-acetoxy-7*β*-hydroxyvouacapan-17*β*-oate	[45]
	Vouacapan-6*α*,7*β*,14*β*,19-tetraol	[46]
	6*α*-Acetoxy-7*β*-hydroxyvouacapane	[9]
	7*β*-Diacetoxyvouacapane	[9]
	6*α*,7*β*-Dihydroxyvouacapan-17*β*-methylene-ol	[9]
	6*α*,7*β*-Dihydroxyvouacapan-17*β*-oate methyl ester	[10]
	6*α*,7*β*-Diacetoxyvouacapane	[47]
	Epoxyfarnesol	[47]
	E-Cariofilene	[48]
	*γ*-Muurolene	[48]
	Bicyclogermacrene	[48]
	6*α*-Acetoxyvouacapane	[48]
	6*α*,7*β*-Dimethoxyvouacapan-17-ene	[48]
	Methyl 6*α*-hydroxy-7*β*-acetoxyvouacapan-17*β*-oate	[14, 15]

Wood	3′,4′,6,7-Tetramethoxyisoflavone	[31]
	2′,6,7-Trimethoxy-4′,5′-methylenedioxyisoflavone	[31]
	2′,3′,4′,6,7-Pentamethoxyisoflavone	[31]

*Pterodon emarginatus*		

Fruits	14,15-Dihydroxy-14,15-dihydrogeranylgeraniol	[49]
	6*α*,7*β*-Diacetoxyvouacapan-14*β*-al	[28]
	6*α*,7*β*-Diacetoxyvouacapan-14*β*-oate	[28]
	Methyl 6*α*,7*β*-diacetoxy-14-hydroxyvinhaticoate	[28]
	Methyl 6*α*,7*β*-diacetoxy-12,16-dihydro-12,14-dihydroxy-16-oxovinhaticoate	[28]
	Trans-caryophyllene	[50]
	*β*-Elemene	[50]
	Germacrene D	[50]
	Spathulenol	[50]
	*α*-Humulene	[50]
	Bicyclogermacrene	[50]
	6*α*,7*β*-Dihydroxyvouacapan-17*β*-oic acid	[51]
	Lupeol	[52]
	14,15-Epoxygeranylgeraniol	[6]
	*β*-Caryophyllene	[12]

Leaves	*γ*-Muurolene	[34]
	Bicyclogermacrene	[34]
	Stigmasterol	[34]
	*β*-Sitosterol	[34]
	Vicenin-2	[35]
	Schaftoside	[35]
	Chrysoeriol-8-*C*-glucosyl-2′′-*O*-glucuronide-6-*C*-arabinoside	[35]
	Luteolin-7-*O*-rutinoside	[35]
	Oleanane-type saponins	[35]
	Hederagenin derivatives	[35]
	Aglycone B	[35]

*Pterodon apparicioi*		

Fruits	7*β*-Acetoxyvouacapane	[28]
	Methyl 6*α*-acetoxy-7*β*-hydroxyvouacapan-17*β*-oate	[28]

*Pterodon polygalaeflorus*		

Fruits	6*α*,7*β*-Dihydroxyvouacapan-17*β*-oic acid	[28]
	Methyl 6*α*,7*β*-dihydroxyvouacapan-17*β*-oate	[28]
	Methyl 6*α*-acetoxy-7*β*-dihydroxyvouacapan-17*β*-oate	[28]
	Vouacapane-6*α*,7*β*,14*β*-triol	[28]
	Methyl 6*α*-acetoxy-7*β*-hydroxyvouacapan-17*β*-oate	[37]
	6*α*-Hydroxy-7*β*-acetoxyvouacap-14(17)-ene	[37]
	6*α*-Hydroxyvouacapane	[1]
	Vouacapane-6*α*,7*β*,14*β*,19-tetraol	[1]
	Taxifolin	[1]
	6*α*-Acetoxyvouacapane	[53]
	Vouacapane	[53]
	Trans-caryophyllene	[54]
	*α*-Copaene	[54]
	*γ*-Muurolene	[54]
	*α*-Humulene	[54]
	Alloaromadendrene	[54]
	Bicyclogermacrene	[54]
	*δ*-Cadinene	[54]
	*α*-Cubenene	[54]
	*γ*-Cadinene	[54]
	*β*-Gurjunene	[54]
	Aromadendrene	[54]
	*α*-Calacorene	[54]
	*δ*-Elemene	[54]
	*α*-Gurjunene	[54]
	*β*-Elemene	[54]
	Spathulenol	[54]
	Germacrene D	[54]
	*β*-Caryophyllene	[4]
	Farnesol	[30]
	Caryophyllene oxide	[30]
	*β*-Humulene	[30]
	Methyl-7*β*-acetoxy-6*α*-hydroxyvouacapan-17*β*-oate	[30]

Wood	6,7-Dimethoxy-3′,4′-methylenodioxyisoflavone	[32]
	3,4,6,7-Tetramethoxyisoflavone	[32]
	4′-Hydroxy-3′,6,7-trimethoxyisoflavone	[32]
	2′,6,7-Trimethoxy-3′,4′-methylenedioxyisoflavone	[32]
	2′,4′,5,6,7-Pentamethoxyisoflavone	[32]
	2′,3′,4′,6,7-Pentamethoxyisoflavone	[32]
	6-Methoxy-7-0-acetyl-3′,4′-Methylenedioxyisoflavone	[32]
	Lupeol	[32]
	Betulin	[32]
	4-Methoxybenzoic	[32]
